# Supplementary material for: Omics Approaches to Assess Flavor Development in Cheese
Source: Foods. 2022 Jan 11;11(2):188. doi: 10.3390/foods11020188 (PMC8775153; doi:10.3390/foods11020188)
Supplement: Supplementary file 1 [file foods-11-00188-s001.zip › foods-1534231-supplementary.pdf]

Supplementary Table S1: Integrative studies combining different omics approaches with respect to cheese flavor formation

Cheese type	Aim of the study	Integrated methods applied	Microorganism/Metabolite	Correlation*	Reference
Fiore Sardo<br>Pecorino Siciliano<br>Pecorino Toscano	Explore the effect of spatial distribution of metabolically active microbiota on secondary proteolysis and VOC production	16S rRNA gene pyrosequencing and PT-GC-MS	*L. plantarum*	2,4-hexadienal, benzaldehyde,<br>methyl and branched esters (+)	[1]
			S. thermophilus	Acetaldehyde, propenal, 2,3-butanedione (+)	
			Lactobacillus sp.	aldehydes (n-alkanals, branched) (+)<br>some primary and secondary alcohols (+)<br>esters (methyl, ethyl, propyl, butyl, 1-methyl-propyl) (+)<br>sulfur compounds (dimethyl sulfide) (+)	
			L. brevis	2,4-hexadienal, 3-heptanone (+)	
			Brevibacterium sp.	14 esters, 9 aldehydes, 2 alcohols, 4 ketones, and 2 sulfur compounds (+)	
			Brachybacterium sp.,<br>*Arthrobacter*,<br>*Halomonas variabilis*	aldehydes (2-heptenal), alcohols (1-hexanol), esters (propyl butanoate), ketones (2,3-octanedione) and sulfur compounds (2-methyl-tetrahydro-thiophen-3-one) (+)	
Caciocavallo Silano	Investigate the effect of increasing ripening temperature on microbial metabolism and	16S rRNA pyrosequencing, shotgun metatranscriptome sequencing and SPME-GC-MS	NSLAB	3-Methyl-1-butanol, volatile short-chain fatty acids (Butanoic acid, Pentanoic acid, Hexanoic acid, Heptanoic acid, Decanoic acid), 2-Nonanone, Nonanal	[2]

	cheese maturation rate				
			L. casei, L. buchneri, L. plantarum, L. gasseri, L. fermentum, L. rhamnosus, Leuconostoc kimchii, Leuc. mesenteroides, Leuc. citreum, Pediococcus pentosaceus	Increased amino acid metabolism activity	
Kazak	Analyze microbiota succession and flavor development during Kazak artisanal cheese ripening	16S V4 / ITS amplicon sequencing and PT–GC-MS	*Acetobacter, Lactococcus, Bacillus, Staphylococcus*	2-nonanone, acetoin and benzaldehyde (+)	[3]
			Escherichia-Shigella, Moraxella	Acetoin (+)	
			Lactobacillus	1-hexanol, 2-heptanone, 2-nonanol, benzyl alcohol, hexanoic acid ethyl ester, nonanoic acid ethyl ester, octanoic acid ethyl ester (-)	
			Kluyveromyces	Butanoic acid, ethyl ester (+)<br>Heptanal (-)	
			Aspergillus	Heptanal, n-decanoic acid (+)	
			Issatchenkia and *Candida*	3-methyl-1-butanol, ethyl acetate, phenylethyl alcohol, 2-methyl-propanoic acid (-)	
			Issatchenkia	n-decanoic acid (+)	
			Candida	Hexanoic acid (+)	
Cheddar cheese curd surface-ripened using commercial	Examine microbial succession and flavor development on the rind in	Whole-metagenome shotgun sequencing and HS-SPME GC-MS	*Debaryomyces hansenii*	Carboxylic acids and alcohols (+)	[4]

smear-culture mixes	surface-ripened cheese				
			Glutamicibacter arilaitensis	3-methyl-1-butanol, phenylethyl alcohol, branched carboxylic acids, 2-heptanol, ketones (+)	
			Brevibacterium linens and *Geotrichum candidum*	Sulfur compounds, 2-methyl-1-butanol (+)	
			Staphylococcus saprophyticus	Ketones, esters, acids, and alcohols (+)	
			Staphylococcus xylosus	Sulfur compounds, 2-methyl-1-butanol, some ethyl esters (+)	
			Corynebacterium variabile	Ketones (+)	
Lab-scale mini-cheese smear-ripened with a culture of *Debaryomyces hansenii*, *Brevibacterium aurantiacum* and *Hafnia alvei*	Characterize metabolic interactions in a microbial synthetic cheese-ripening community	Microbial enumeration, UHPLC-MS, HPLC-UV, dual RNA-seq	*H. alvei*	Encodes a gene cluster for siderophore biosynthesis, increasing thus iron availability for *B. aurantiacum*<br>Increases sulfur amino acid availability for *B. aurantiacum*	[5]
			B. aurantiacum	Lipolysis of triglycerides from *B. aurantiacum* increases glycerol concentration in cheese, serving as energy substrate for *H. alvei*<br>Proteolysis of caseins from *B. aurantiacum* increases nitrogen availability for *D. hansenii* and *H. alvei,* while proteolytic activity of *B. aurantiacum* and other cheese microorganisms increased methionine catabolism in *H. alvei*, producing methanethiol	
Macrococcus caseolyticus subsp. *caseolyticus*	Explore the metabolic potential of *Macrococcus caseolyticus* subsp.	Whole genome sequencing and HS-SPME GC-MS	*Macrococcus caseolyticus* subsp. *caseolyticus* strains of dairy and non-dairy origin	Analysis of proteolytic and lipolytic systems components, strains' volatile profile when grown in lactose-free milk, association with FFA metabolism	[6]

	caseolyticus to contribute to flavor formation				
Artisanal and industrial cheddar cheese	Determine microbiota-metabolome relationships of artisanal and industrial cheddar cheeses	16S rRNA amplicon sequencing, untargeted GC-MS, and LC-MS metabolomics	*Macrococcus*	13 metabolites including two dipeptides and proline, in industrial cheeses (surface)	[7]
			Lactobacillus	Asparagine, proline, lysine, asparagine-valine, in industrial cheeses	
			Pediococcus	21 metabolites including 1-formyl-pentadioic acid, a branched chain fatty acid (BCFA), in artisanal cheeses	
			Streptococcus and *Lactobacillus*	3-hydroxy propanoic acid **only** in artisanal cheeses (+)	
			Streptococcus	O-Methoxycatechol O-sulphate in artisanal cheeses (surface) (+)	
			Streptococcus and *Lactobacillus*	5-amino valeric acid, GABA, tyramine, in artisanal cheeses (core and surface)	
Commercial industrial cheddar cheeses of different ripening ages made by different manufacturers	Identify key microbiota and/or metabolite profiles of retail industrial cheddar cheeses of varying maturity and determine microbiota - metabolites interrelationships	16S rRNA amplicon sequencing, and untargeted GC-MS metabolomics	*Thermus* in brand A	Phenylalanine (+)	[8]
			Streptococcus in brand B	Urea and lactose (-)	
			Streptococcus in brand C	Cholesterol and urea (-)	

			Lactococcus in brand B	Heptadecanoic acid (+)<br>Urea (+)	
			Lactococcus in brand C	Pyroglutamic acid and piperidine (-)	
			Lactobacillus in brand C	Tyrosine (+)<br>Ornithine and glutamine (-)	
Similar-style cheddar cheeses of different qualities made by the same manufacturer	Determine microbiota and/or metabolite signatures (biomarkers) that could be used to assess cheese quality	16S rRNA amplicon sequencing, and untargeted GC-MS metabolomics	*Streptococcus*<br>*L. lactis*	High-quality cheeses (+)<br>Low-quality cheeses (+)	[9]
			Streptococcus in high-quality cheeses	Aspartic acid, isoleucine, histidine, and proline (+)	
			L. lactis in low-quality cheeses	Stearic acid and octadecanol (+)	
Soft, semi-hard, and hard artisanal cheeses from Ireland together with publicly available cheese metagenomes	Assess the contribution of cheese microbiota to cheese quality	Meta-analysis of cheese microbiomes and corresponding volatilomes by GC-MS	Strain-level variation in species of *Brevibacterium linens*, *L. lactis* and *S. thermophilus*	Variations in volatile levels	[10]
			Metagenome-assembled genomes from putatively novel species	Acetate, succinate, lactate, and ammonium (+)	
Cheese curd agar inoculated with strains of the same three bacterial species, isolated from cheese rinds	Elucidate the impact of strain diversity across microbiomes from distinct geographic regions on community	Comparative genomics and HSSE-GC-MS	Cheese communities consisting of different strains of the same three species	Differing responses to abiotic (high salt) and biotic (the fungus *Penicillium*) perturbations, as well as variation in composition of VOCs emitted	[11]

of different origin to construct synthetic microbial communities that mimic the microbial dynamics and functions of real surface-ripened cheese rinds	assembly dynamics and functional				
362 cheese rind samples collected from 137 different cheese types	Evaluate cheese microbiota diversity and functional potential	Amplicon and shotgun metagenomic sequencing	14 bacterial and 10 fungal genera	Rind type (natural, washed, or bloomy)	[12]
			-	Genes involved in several metabolic pathways associated with flavor formation	
			Pseudoalteromonas spp.	Cold-adapted enzymes that participate in lipolysis and proteolysis	
Experimental surface-ripened cheese composed of nine microbial species	Explore the dynamics of the cheese microbial community, the metabolic activities of the different community members and their possible interactions during cheese ripening	Metagenomic and metatranscriptomic analysis	*L. lactis* and *K. lactis*	Expression of enzymes involved in lactose fermentation (+)	[13]
			D. hansenii and *G. candidum*	High levels of lactate dehydrogenase transcripts (+)	

			G. candidum	Transcripts for proteolysis, lipolysis, and amino acid catabolism (+)	
Maasdam cheese ripened in different temperatures	Cheese microbiota characterization and gene expression during warm and cold room ripening	Metagenomic and metatranscriptomic analysis	*L. lactis* (subp. *lactis* or *cremoris*)	Genes for FFA biosynthesis, proteolysis, lipolysis, valine degradation and methionine-cysteine degradation (sulfur containing amino acids)	[14]
			L. rhamnosus	Genes for FFA biosynthesis, proteolysis, lipolysis, and methionine-cysteine degradation (sulfur containing amino acids)	
			L. helveticus	Genes for FFA biosynthesis, proteolysis, and methionine-cysteine degradation (sulfur containing amino acids)	
			P. freudenreichii subsp. *shermanii*	Genes for FFA biosynthesis, lipolysis, valine degradation and methionine-cysteine degradation (sulfur containing amino acids	
Historic Rebel cheese	Assess the biodiversity and richness of bacterial communities, the volatilome, terpenes and fatty acid profiles in HR cheese in order to characterize it	16S rRNA gene sequencing, volatilome analysis by SPME GC-MS, terpene fraction and FA by Dynamic Headspace-Gas Chromatography-Mass Spectrometry (DHS-GC-MS)	*Lactococcus*	3-Hydroxybutan-2-one, acetoin (+)	[15]
			Leuconostoc	Most ethyl-esters, ethanol, propionic acid (+)	
			Lactobacillus	2-Heptanol (+)	
			Streptococcus	Hexanal, 1-hexanol (+)	
Pélardon cheese	Unravel bacterial and fungal dynamics during cheese	16S rRNA and ITS gene sequencing. Volatile analysis by HS GC–MS	*L. lactis* and *L. mesenteroides*	Lactic acid, acetic acid, and 3-hydroxybutan-2-one (+)	[16]

	preparation and ripening and determine their contribution in aroma production		
		L. mesenteroides	3-methylbutanoic and 2-methylpropanoic acid (+)
		G. candidum	2-phenylacetaldehyde and 2-phenylethanol (+)
		L. paracasei, E. faecalis, P. commune, S. brevicaulis	Ketones originating from FFA catabolism, responsible for strong “earthy” and “cheesy” notes

*(+): Positive correlation; (-): Negative correlation

References

1. De Pasquale, I.; Di Cagno, R.; Buchin, S.; De Angelis, M.; Gobbetti, M. Spatial distribution of the metabolically active microbiota within Italian PDO ewes' milk cheeses. *PLOS ONE*, **2016**, *11*, e0153213. DOI: 10.1371/journal.pone.0153213.
2. De Filippis, F.; Genovese, A.; Ferranti, P.; Gilbert, J.A.; Ercolini, D. Metatranscriptomics reveals temperature-driven functional changes in microbiome impacting cheese maturation rate. *Sci. Rep.* **2016**, *6*, 21871. DOI: 10.1038/srep21871.
3. Zheng, X.; Liu, F.; Shi, X.; Wang, B.; Li, K.; Li, B.; Zhuge, B. Dynamic correlations between microbiota succession and flavor development involved in the ripening of Kazak artisanal cheese. *Food Res. Int.* **2018**, *105*, 733-742. DOI: 10.1016/j.foodres.2017.12.007.
4. Bertuzzi, A.S.; Walsh, A.M.; Sheehan, J.J.; Cotter, P.D.; Crispie, F.; McSweeney, P.L.H.; Kilcawley, K.N.; Rea, M.C. Omics-based insights into flavor development and microbial succession within surface-ripened cheese. *mSystems* **2018**, *3*, e00211-00217. DOI: 10.1128/mSystems.00211-17.
5. Pham, N.-P.; Landaud, S.; Lieben, P.; Bonnarme, P.; Monnet, C. Transcription profiling reveals cooperative metabolic interactions in a microbial cheese-ripening community composed of *Debaryomyces hansenii, Brevibacterium aurantiacum*, and *Hafnia alvei. Front. microbiol.* **2019**, *10*, DOI: 10.3389/fmicb.2019.01901.
6. Mazhar, S.; Kilcawley, K.N.; Hill, C.; McAuliffe, O. A systems-wide analysis of proteolytic and lipolytic pathways uncovers the flavor-forming potential of the gram-positive bacterium *Macrococcus caseolyticus* subsp. *caseolyticus. Front. microbiol.* **2020**, *11*, DOI: 10.3389/fmicb.2020.01533.
7. Afshari, R.; Pillidge, C.J.; Read, E.; Rochfort, S.; Dias, D.A.; Osborn, A.M.; Gill, H. New insights into cheddar cheese microbiota-metabolome relationships revealed by integrative analysis of multi-omics data. *Sci. Rep.* **2020**, *10*, 3164. DOI: 10.1038/s41598-020-59617-9.
8. Afshari, R.; Pillidge, C.J.; Dias, D.A.; Osborn, A.M.; Gill, H. Microbiota and metabolite profiling combined with integrative analysis for differentiating cheeses of varying ripening ages. *Front. Microbiol.* **2020**, *11*, 2991. DOI: 10.3389/fmicb.2020.592060.
9. Afshari, R.; Pillidge, C.J.; Dias, D.A.; Osborn, A.M.; Gill, H. Biomarkers associated with cheese quality uncovered by integrative multi-omic analysis. *Food Control* **2021**, *123*, 107752. https://doi.org/10.1016/j.foodcont.2020.107752.
10. Walsh, A.M.; Macori, G.; Kilcawley, K.N.; Cotter, P.D. Meta-analysis of cheese microbiomes highlights contributions to multiple aspects of quality. *Nature Food* **2020**, *1*, 500-510. DOI: 10.1038/s43016-020-0129-3.

11. Niccum Brittany, A.; Kastman Erik, K.; Kfoury, N.; Robbat, A.; Wolfe Benjamin, E.; Cotter Paul, D. Strain-level diversity impacts cheese rind microbiome assembly and function. *mSystems*, **2020**, *5*, e00149-00120. DOI: 10.1128/mSystems.00149-20.
12. Wolfe, B.E.; Button, J.E.; Santarelli, M.; Dutton, R.J. Cheese rind communities provide tractable systems for in situ and in vitro studies of microbial diversity. *Cell* **2014**, *158*, 422-433. DOI: 10.1016/j.cell.2014.05.041.
13. Dugat-Bony, E.; Straub, C.; Teissandier, A.; Onésime, D.; Loux, V.; Monnet, C.; Irlinger, F.; Landaud, S.; Leclercq-Perlat, M.-N.; Bento, P.; Fraud, S.; Gibrat, J.-F.; Aubert, J.; Fer, F.; Guédon, E.; Pons, N.; Kennedy, S.; Beckerich, J.-M.; Swennen, D.; Bonnarme, P. Overview of a surface-ripened cheese community functioning by meta-omics analyses. *PLOS ONE*, **2015**, *10*, e0124360. DOI: 10.1371/journal.pone.0124360.
14. Duru, I.C.; Laine, P.; Andreevskaya, M.; Paulin, L.; Kananen, S.; Tynkkynen, S.; Auvinen, P.; Smolander, O.P. Metagenomic and metatranscriptomic analysis of the microbial community in Swiss-type Maasdam cheese during ripening. *Int. J. Food Microbiol.* **2018**, *281*, 10-22. DOI: 10.1016/j.ijfoodmicro.2018.05.017.
15. Turri, F.; Cremonesi, P.; Battelli, G.; Severgnini, M.; Brasca, M.; Gandini, G.; Pizzi, F. High biodiversity in a limited mountain area revealed in the traditional production of Historic Rebel cheese by an integrated microbiota–lipidomic approach. *Sci. Rep.* **2021**, *11*, 10374. DOI: 10.1038/s41598-021-89959-x.
16. Penland, M.; Falentin, H.; Parayre, S.; Pawtowski, A.; Maillard, M.-B.; Thierry, A.; Mounier, J.; Coton, M.; Deutsch, S.-M. Linking Pélardon artisanal goat cheese microbial communities to aroma compounds during cheese-making and ripening. *Int. J. Food Microbiol.* **2021**, *345*, 109130. https://doi.org/10.1016/j.ijfoodmicro.2021.109130.
